# Expression of 5S rDNA in the oocytes of water frogs

**DOI:** 10.1186/1756-0500-2-10

**Published:** 2009-01-19

**Authors:** Elżbieta Czarniewska, Robert Plewa

**Affiliations:** 1 Department of Animal Physiology and Development, Institute of Experimental Biology, Adam Mickiewicz University, Umultowska Str. 89, 61-614 Poznañ, Poland

## Abstract

**Background:**

We report the expression pattern of *5S rDNA *in the eggs of water frogs *Rana lessonae*, *Rana ridibunda *and *Rana esculenta *using the quantitative real-time PCR. This kind of research had never been performed before.

**Results:**

*5S rDNA *relative expression of the *Rana ridibunda *oocytes is approximately six times higher in comparison to the *Rana lessonae *oocytes. The oocytes of the investigated *Rana esculenta *frogs, in respect of *5S rDNA *relative expression ratio, were very similar to the *Rana ridibunda *oocytes.

**Conclusion:**

We suggest the possibility of using *5S rDNA *as the internal control gene, in the studies of relative mRNA quantitative assays in water frog oocytes, because of its characteristic specific expression pattern in the *Rana lessonae*, *Rana ridibunda *and *Rana esculenta *oocytes.

## Findings

An amphibian egg contains all the information required for its early post-fertilisation proliferation and differentiation. During the initial stages of development, all translated mRNAs, as well as ribosomes – the cellular organelles responsible for protein biosynthesis – originate from the mother. They play a crucial role in the success of early embryonic development, allowing the first cleavages to occur, before the activation of embryonic genome after the midblastula transition (MBT) [1,2].

The eucaryotic ribosome is a macromolecular structure composed of a large (60S) and a small (40S) subunits. Biochemically, it is composed of four ribosomal RNA molecules (rRNAs) and over 70 ribosomal proteins [3]. In eukaryotes, two distinct classes of ribosomal DNA (*rDNA*) genes can be distinguished. Each is composed of tandemly repeated units of hundreds to thousands of copies. The transcripts of the minor class (5S rRNA) are made from one region of the genome, the transcripts of 28S, 18S and 5.8S rRNAs (the major class; *45S rDNA*) from another. The *5S rDNA *array consists of multiple copies of a highly conserved 120 bp coding sequence, which are separated by the variable nontranscribed spacers (NTS) [4]. The *Xenopus *genome contains two sets of *5S rDNA*: the oocyte-type, active only in oocytes (20,000 tandemly-repeated copies per haploid genome), and the somatic-type, active in every cell (400 copies per haploid genome). The synthesis of ribosomal RNAs in amphibian oocytes takes place during the prolonged diplotene stage of the first meiotic division [5].

*Rana esculenta *(genotype RL), a common water frog of Europe, is not a conventional species. It arises by the hybridisation between two Mendelian species: *Rana lessonae *(genotype LL) and *Rana ridibunda *(genotype RR), and normally reproduces by hybridogenesis [6,7]. Prior to meiosis, the lessonae genome is eliminated, so that RL females produce eggs that contain only the ridibunda genome. The somatic hybridity of RL is restored in the offspring through mating with LL [7]. Until now, however, the mechanism of hybridogenesis in RL is not known.

Quantitative real-time RT PCR (Q-PCR) is an established method for quantifying mRNA in biological samples. To analyse the expression level of a particular gene, its level of expression is compared to the expression of a reference gene. For this to be effective, the reference genes should be expressed at a constant level in certain tissues, at all stages of development, throughout the cell cycle, and without effects caused by experimental treatment. To date, the most commonly used reference genes include β-actin, glyceraldehyde-3-phosphate dehydrogenase (GAPDH), hypoxantine-guanine phosphoribosyl transferase (HPRT) and 18S rRNA [8,9]. Unfortunately, several recent studies have shown that the expression of reference genes differed depending on kind of tissues, developmental stages and experimental conditions [8,9].

**Figure 1:**
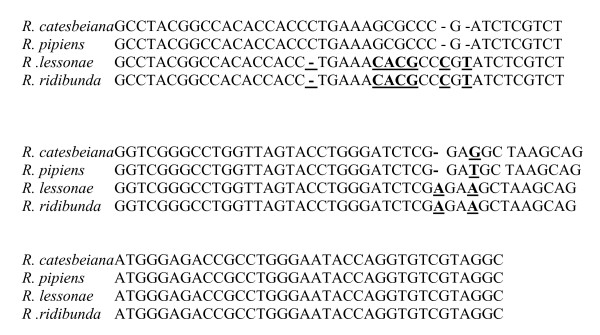
**Comparison of the nucleotide sequence of *5S rDNA *between the *R. lessonae*, *R. ridibunda*, *R. catesbeiana and R. pipiens***.

**Figure 2:**
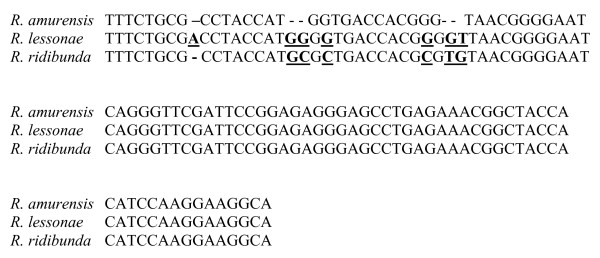
**Comparison of the nucleotide sequence of *18S rDNA *fragment between the *R. lessonae*, *R. ridibunda *and *R. amurensis***.

In the present study, for the first time in the research on amphibians, we examined the expression level of *5S rDNA *in oocytes of water frogs. We used *18S rDNA *as a reference gene. The specificity of the RT-PCR products was confirmed by sequencing reactions. Alignment of the *5S rDNA *sequences of LL, RR, *R. catesbeiana *([GenBank:X58367]; [10]) and *R. pipiens *([GenBank:X58368]; [10]) is shown in Fig. 1, whereas comparison of a highly conserved region of the *18S rDNA *of LL, RR and *R. amurensis *([GenBank:AF542043.]; [11]) is shown in Fig. 2. The analysed *5S rDNA *of LL ([GenBank:FJ572051]) and RR ([GenBank:FJ572052]) showed 95% and 93% sequence homology to the respective *5S rDNA *of *R. catesbeiana *and *R. pipiens*. The LL ([GenBank:FJ572053]) and RR ([GenBank:FJ572054]) *18S rDNA *nucleotide sequences showed 96% and 93% sequence identity to *R. amurensis 18S rDNA*, respectively.

By using Q-PCR, we analysed the relative expression of *5S rDNA*. Each sample was first normalized according to the amount of template added and to the expression level of the endogenous control *18S rDNA*. The obtained values were further normalized with relation to a calibrator. In our studies, we used LL rather than RR as the calibrator because in nature, LL arises only from the LL x LL matings, whereas RR can arise from RR x RR, RR x RL and RL x RL matings. Usually, RL x RL matings lead to inviable offspring, probably because they are homozygous for deleterious mutations in the clonal ridibunda genomes [12,13], although occasionally RL x RL matings lead to some viable offspring and fertile RR females.

**Figure 3:**
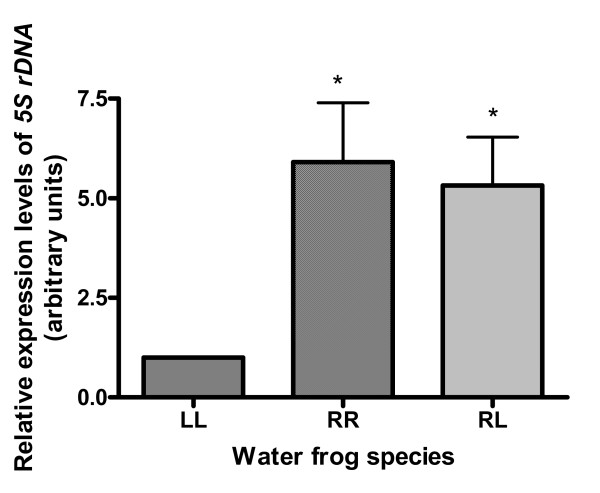
**Relative expression of *5S rDNA *in LL, RR and RL oocytes**. The error bars represent SEM (* statistically significant); p < 0.05. LL – *R. lessonae*, RR – *R. ridibunda*, RL – *R. esculenta*.

The expression levels of *5S rDNA *in the oocytes of the two Mendelian species are significantly different, approximately six times higher in RR oocytes than in LL oocytes. 5S rDNA expression in oocytes of RL frogs investigated, was similar to that of RR oocytes and much higher than that of LL oocytes (Fig. 3).

It is not surprising, that the oocytes produced by RL are similar to the RR oocytes. The lessonae chromosome set is absent in the RL ova, which contain only the ridibunda chromosome set [7]. It is expected that in the RL oocytes, as a result of hybridogenetic mode of the RL reproduction, the ridibunda characteristic pattern of the gene expression should be restored. Our research confirmed that fact.

Behavioural studies and field observations indicate that the hybridisation that produces RL always occurs between RR females and LL males [15,16]. This directionality of interspecific mating depends on the body size preferences shown by males. The combination of a large female with a small male is strongly preferred in water frogs [16]. Both in nature and in laboratory conditions, males consistently avoid females smaller than themselves, so the opposite mating is not observed. Similarly to RR females, the RL females also preferentially reproduce via hybridogenesis with smaller LL males [16]. The RL oocytes contain the unrecombined ridibunda genome, and those proteins that have been investigated in oocytes are of the ridibunda type [17,18]. Our data demonstrate that the RL eggs, in respect of the *5S rDNA *expression, also resemble the ridibunda type.

It is generally accepted that a carefully selected stable internal control gene should normalize the gene-expression level. To find a true reference gene with a constant and stable transcription in specific tissue or cells would be extremely useful, but such a gene has not been identified yet [9]. The results of our study suggest the possibility of using *5S rDNA *as the internal control gene in relative mRNA quantitative assays in LL, RR and RL eggs, because they have the specific expression levels.

## Conclusion

The oocytes of two Mendelian species (LL and RR) differ from each other with respect to the relative expression level of *5S rDNA*. The oocytes of the hybridogenetic RL species are similar to the RR eggs in level of *5S rDNA *expression, which results from the RL hybridogenetic mode of reproduction.

## Methods

All procedures were approved by the Local Ethical Committee on Experiments on Animals in Poznañ, permit No. 51/2006.

### Oocytes

Frogs oocytes were obtained from LL, RR and RL by the routine *in vitro *method [19]. Ovulation was induced by using the salmon Luteinizing Hormone – Releasing Hormone (LHRH; H-7525, Bachem Bioscience Inc.), which was injected into the lymph sacs in the doses of approximately 1 μg/10 g body weight. Ovulation usually occurs within 24 hours, although, occasionally a second injection is necessary.

### RNA extractions and cDNA syntheses

Total RNA was isolated from 10 oocytes/1 sample using TRI Reagent (Sigma) according to the manufacturer's isolation instructions. To purify the RNA probe from genomic DNA contamination, we treated the samples with RNase-free DNAse I (Fermentas), according to the manufacturer's instructions. The quality of RNA was checked on agarose gel.

1 μg of total RNA was reverse transcribed using 3 μl of specific primers in water in a total volume of 11 μl. The mixture was incubated for 5 min at 70°C. After cooling on ice, 4 μl of reverse transcription buffer (5×), 2 μl of 10 mM dNTPs, and DEPC-treated water to the volume of 19 μl were added. Later, the mixture was incubated for 5 min at 37°C. After this time, 200 U of RevertAid M-MuLV Reverse Transcriptase (Fermentas) was added, and the reaction mixture was incubated for 60 min at 42°C. As negative controls, the mixtures without RNA were prepared.

The specific primers used in the RT-PCR, sequencing and Q-PCR reactions were:

*- 5S rDNA*: 5'-GCCTACGGCCACACCACC-3' and 5'-AAGCCTACGACACCTGGTAT-3'

*- 18S rDNA*: 5'-CGTCTGCCCTATCAACTTTCG-3' and 5'-TGCCTTCCTTGGATGTGGTAG-3'.

The *18S rDNA *primers were designed based on the nucleotide sequence determined for region of *18S rDNA *(from 353 nt to 448 nt) of *Rana amurensis *([GenBank:AF542043.]; [11]).

### Sequencing reaction

The RT-PCR products were sequenced on a Seq CEQ™ 8000 Genetic Analysis System (Beckman Coulter) with a Genome Lab™ DTCS – Quick Start (Beckman Coulter), according to the manufacturer's instructions. The sequences of the amplification products were compared to the GenBank sequences with the BLAST sequences software .

### Quantitative RT-PCR

The Q-PCR analysis was performed using the Finzymes's DyNAmo™ HS SYBR^® ^Green qPCR according to the manufacturer's instructions. The reaction mixture, in the final volume of 20 μl, contained 5 μl of cDNA (500× diluted), 10 μl of master mix, 0.5 mM of forward and reverse specific primer, 0.2 μl of ROX, and 2,8 μl of H_2_O. The analysis was carried out on the Rotor-Gene™ 6000 (Corbett Life Science) with cycle conditions: initial 15 min of denaturation and enzyme activation at 94°C, 40 cycles each of 94°C for 10 sec, 58°C for 15 sec and 72°C for 30 sec. A melt curve was produced to confirm a single-specific peak and to detect primer/dimmer formation by heating the samples by 0.5°C increments from 72 to 95°C, with a dwell time at each temperature for 10 seconds. During that time, the continuous monitoring of the fluorescence was being performed.

For the relative quantification of the *5S rDNA *expression in the LL, RR and RL oocytes, the ΔΔCt-method was used [20].

### Statistical analysis

Results were expressed as the mean ± SEM. Significance was accepted for p < 0.05. Data were analysed using GraphPad (Prism 4) software.

## Competing interests

The authors declare that they have no competing interests.

## Authors' contributions

EC conceived of the study, carried out the main body of the project and prepared the manuscript. RP participated in the sequencing of the RT-PCR products and provided support throughout the experimental process. Both authors read and approved the final manuscript.
